# Performances of novel custom 3D-printed cutting guide in canine caudal maxillectomy: a cadaveric study

**DOI:** 10.3389/fvets.2023.1127025

**Published:** 2023-06-08

**Authors:** Aidan Chambers, Marine Traverson, Shelby Neal, Satyanarayana Konala, Ola Harrysson

**Affiliations:** ^1^Department of Clinical Sciences, College of Veterinary Medicine, North Carolina State University, Raleigh, NC, United States; ^2^Center for Additive Manufacturing and Logistics, College of Engineering, North Carolina State University, Raleigh, NC, United States; ^3^Department of Chemical and Biomolecular Engineering, North Carolina State University, Raleigh, NC, United States; ^4^Fitts Department of Industrial and Systems Engineering, North Carolina State University, Raleigh, NC, United States; ^5^Department of Biomedical Engineering, College of Engineering, North Carolina State University, Raleigh, NC, United States; ^6^Department of Material Science and Engineering, College of Engineering, North Carolina State University, Raleigh, NC, United States

**Keywords:** 3D-printing, maxillectomy, surgical guide, accuracy, oncologic margins

## Abstract

**Introduction:**

Caudal maxillectomies are challenging procedures for most veterinary surgeons. Custom guides may allow the procedure to become more accessible.

**Methods:**

A cadaveric study was performed to evaluate the accuracy and efficiency of stereolithography guided (3D-printed) caudal maxillectomy. Mean absolute linear deviation from planned to performed cuts and mean procedure duration were compared pairwise between three study groups, with 10 canine cadaver head sides per group: 3D-printed guided caudal maxillectomy performed by an experienced surgeon (ESG) and a novice surgery resident (NSG), and freehand procedure performed by an experienced surgeon (ESF).

**Results:**

Accuracy was systematically higher for ESG versus ESF, and statistically significant for 4 of 5 osteotomies (*p* < 0.05). There was no statistical difference in accuracy between ESG and NSG. The highest absolute mean linear deviation for ESG was <2 mm and >5 mm for ESF. Procedure duration was statistically significantly longer for ESG than ESF (*p* < 0.001), and for NSG than ESG (*p* < 0.001).

**Discussion:**

Surgical accuracy of canine caudal maxillectomy was improved with the use of our novel custom cutting guide, despite a longer duration procedure. Improved accuracy obtained with the use of the custom cutting guide could prove beneficial in achieving complete oncologic margins. The time increase might be acceptable if hemorrhage can be adequately controlled *in vivo*. Further development in custom guides may improve the overall efficacy of the procedure.

## 1. Introduction

Tumors of the oral cavity make up approximately 6% of all tumors in dogs, with malignant melanoma, squamous cell carcinoma, and fibrosarcoma being the most common neoplasms. Wide surgical excision with 1–2 cm gross surgical margins is recommended to obtain local tumor control in malignant cases and locally invasive benign cases such as acanthomatous ameloblastoma (1, 2). MacLellan et al. (2), however, reported that 31.6% of dogs undergoing any type of partial maxillectomy had incomplete histologic margins. Incomplete resection has been intimately associated with local tumor recurrence, and 65% of maxillary tumors removed with incomplete margins have been reported to recur locally versus 22% of tumors removed with complete margins (2–4). Anatomic location has also been proven to impact local tumor control. Sarowitz et al. (3) found that caudal maxillary tumors were associated with a 1.5 times hazard ratio for local tumor recurrence compared to all other maxillary tumors. This is potentially related to the difficulty of obtaining complete histologic margins in this challenging anatomic location (3–5). Indeed, caudal maxillary tumors are believed to be difficult to remove because of poorer surgical exposure compared to rostral tumors, more abundant vasculature caudally, and generally larger size masses that have escaped owners’ notice until they start causing clinical signs (3–5). There is a need to increase technical ease and intraoperative accuracy for the excision of caudal maxillary tumors to optimize oncologic margins and improve long-term local tumor control.

Stereolithography (3D-printing) is becoming more popular in human and veterinary surgery because of the ability to make custom surgical guides, patient specific anatomical models, and individualized prostheses based on the diagnostic images of the patient (6–10). Stereolithography allows three dimensional models to be created in a variety of polymer materials using a laser light source that selectively cures and solidifies a liquid plastic layer-by-layer along the cross-section of the object (8, 10). Custom-made drilling and cutting guides have been demonstrated to aid in surgical accuracy, efficiency, and allow implants to be best fitted to the patient (9, 10). In the human surgical field, 3D-printed surgical guides have been largely used to improve accuracy in dental procedures with small margins of error and to maximize the chances of obtaining clean surgical margins in oncologic procedures (11–13). The use of a custom surgical guide has been shown to improve accuracy in 76%–92% of cases compared to traditional freehand procedures for osteotomies and complex reconstructions in human maxillofacial, dental, orthopedic, and oncologic surgery (7, 9). In addition to the maxilla being a complex surgical location, there is great variability in anatomy between breeds within the canine species (14, 15). Therefore, the use of 3D-printed custom-made surgical guides may also improve accuracy and local tumor control for canine caudal maxillectomy procedures.

Additionally, major surgical hemorrhage has been reported as the number one intraoperative complication in canine maxillectomy, and occurred in 83% of dogs undergoing a caudal maxillectomy *via* a combined dorsolateral and intraoral approach in a retrospective study by MacLellan et al. (2). As a consequence, patients undergoing a complete or caudal maxillectomy are reportedly 3 to 6.5 times more at risk of requiring a blood transfusion intraoperatively compared to other types of maxillectomy and/or mandibulectomy procedures (2, 16). This is thought to be related to the complexity of the locoregional vasculature (2, 16). Additionally, median duration of the surgical procedure is also significantly increased for a caudal maxillectomy compared to other types of maxillectomies, and certainly plays a role in the increased hemorrhagic risk (2). In addition to improvements in accuracy, the use of a 3D-printed cutting and drilling guide created by computer aided manufacturing (CAM) has been shown to decrease the amount of time spent in the operating room (7, 9). In a systematic review of 227 human surgical studies using 3D-printing technology, 28% of reports using 3D-printed custom-made surgical guides saw a reduction in operating room or treatment time with the use of CAM compared to conventional planning methods (7). The use of a 3D-printed custom-made surgical guide may decrease the duration of caudal maxillectomy procedures and allow for more rapid hemostasis to be obtained, lowering the morbidity associated with the surgery.

Finally, the use of a 3D-printed surgical guide has the potential to not only improve the efficiency and accuracy of caudal maxillectomy procedures performed by experimented surgeons, but also to make these complex procedures more accessible to novice surgeons or boarded surgeons with limited experience in caudal maxillectomy. In the human literature, there is conflicting information regarding the use of 3D-printed surgical guides and whether such guides facilitate the procedures in the hands of a novice surgeon in comparison to an experienced surgeon (8, 11–13). Because of the wide range of procedures that veterinary surgeons are trained to do, it is possible for a residency-trained surgeon to not have primary experience with a caudal maxillectomy by the time they complete their program. The use of a 3D-printed cutting guide, however, may allow a caudal maxillectomy to become more accessible to veterinary surgeons that are advanced in their surgical training but have not yet had the opportunity to perform such a procedure. Novice surgeons also have a tendency to perform procedures slower than experienced surgeons; that difference is even more pronounced with complex procedures, as demonstrated in human surgery (17, 18). For a complex procedure such as a caudal maxillectomy, the use of a 3D-printed custom-made cutting guide may allow a novice surgeon to perform the surgery in a duration comparable to an experienced surgeon.

The objectives of this study were to (1) design a 3D-printed custom-made caudal maxillectomy surgical guide, and to (2) evaluate the accuracy and efficiency of the surgical guide in cadaveric dogs. We hypothesized that the use of a 3D-printed custom-made surgical guide increases the accuracy and efficiency of the osteotomy compared to a standard freehand procedure (hypothesis 1), and that there would not be any difference in accuracy and efficiency between a novice and an experienced surgeon when using the cutting guide (hypothesis 2).

## 2. Materials and methods

### 2.1. Specimen randomization

Fifteen heads were obtained from fresh frozen canine cadavers euthanatized at local shelters for reasons unrelated to the study and thawed in preparation to the procedure. Both left and right sides of the heads were used as separate subjects. Head number and lateralization were randomized per testing group and order with 10 head sides per treatment group. The heads were inspected for any visual abnormality, and classified as doli-, brachy-, or mesocephalic. Their length was recorded from the most ventral aspect of the maxillary canine to the caudal aspect of the occipital bone.

### 2.2. Study groups

**Figure 1:**
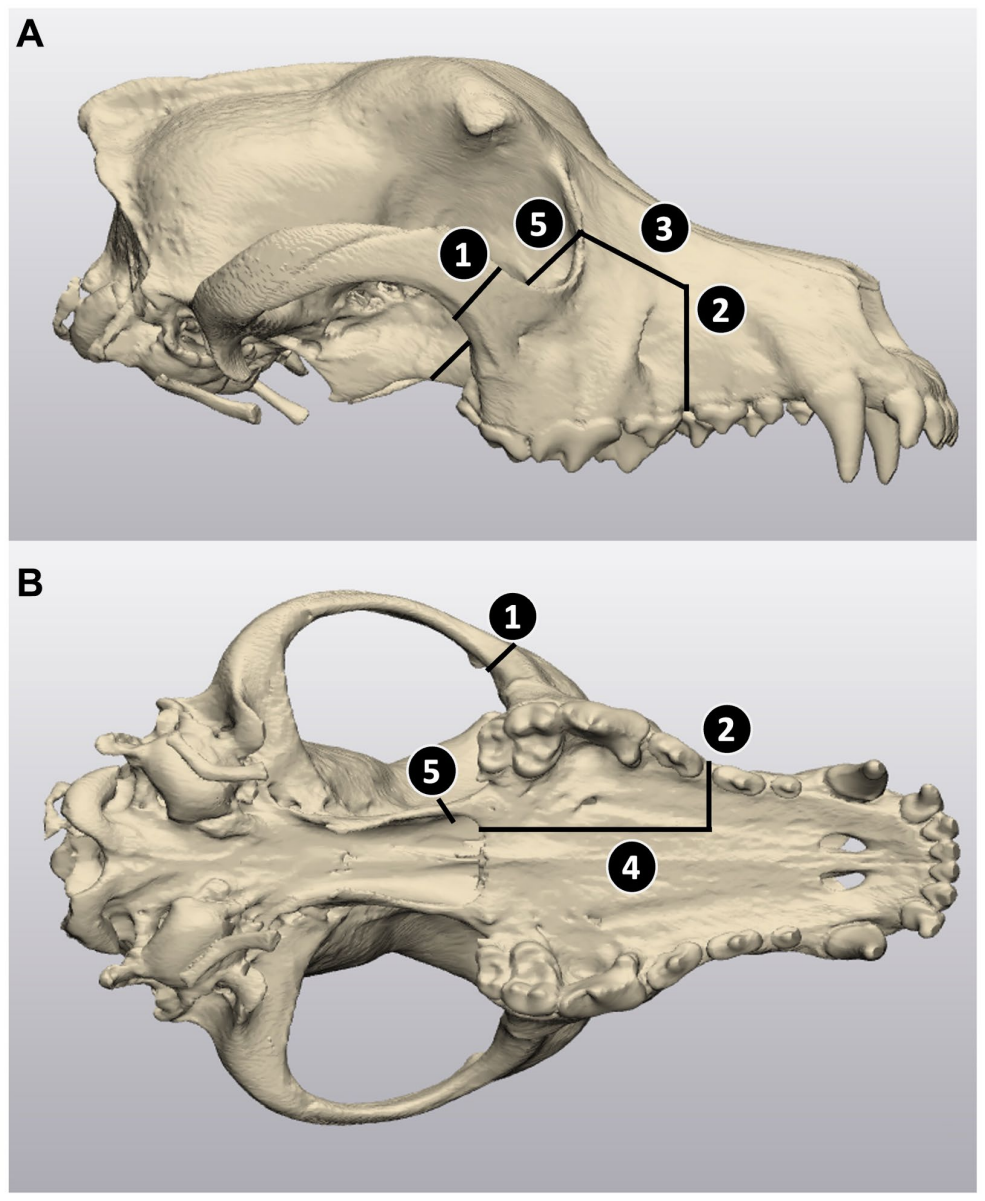
Virtual caudal maxillectomy planning in 3-Matic. **(A)** Lateral view of the skull; **(B)** ventral intraoral view. The cuts for the maxillectomies were planned based on the following anatomical landmarks: (1) zygomatic cut, located caudal to the zygomaticomaxillary suture, (2) rostrolateral cut, extending to one third of the width of the palate at the rostral aspect of the third maxillary premolar tooth, to 5 mm rostral and dorsal to the infraorbital foramen, (3) dorsolateral cut, joining the dorsolateral extremity of cut 2 to the mid orbital rim of the lacrimal bone, (4) palatine cut, extending from the rostral intra-oral extremity of cut 2 to the caudal edge of the hard palate along the third of the width of the palate (5) orbit cut, angled from the extremity of cut 3 at the orbital rim to the medial aspect of the second maxillary molar tooth.

Two experimenters participated in this study: a board-certified surgeon and surgical oncologist (MT) with experience using 3D-printed guided implantology (considered the experienced surgeon) while the other experimenter (AC) was a second-year surgical resident at the time of the experimentation (considered the novice surgeon). To address the study objectives, three treatment groups were used that consisted of caudal maxillectomy procedures performed on canine cadaver heads (1) with the use of individualized 3D-printed surgical guide by the experienced surgeon (group ESG), (2) with the use of individualized 3D-printed surgical guide by the novice surgeon (group NSG), or (3) freehand by the experienced surgeon (group ESF). Both the experienced and novice surgeons were right hand dominant and assisted to the other’s procedures (see Figure 1).

### 2.3. Maxillectomy planning

Computed tomography (CT) scans of all cadaver heads were performed *via* a 64-slice CT scanner (Siemens 64 slice; Siemens Medical Solutions, Malvern, Pennsylvania) and imported into a DICOM image processing software (Mimics Innovation Suite, Materialise, Leuven, Belgium). Following the procedure described by Lascelles et al. (19), five osteotomy cuts were defined to complete a caudal maxillectomy with ventral orbitectomy, (1) zygomatic cut, (2) rostrolateral cut, (3) dorsolateral cut, (4) palatine cut, and (5) orbital rim cut. The skulls were thresholded, segmented, and virtual osteotomy cuts were planned in 3-Matics software (Mimics Innovation Suite, Materialise, Leuven, Belgium) (see Figure 1).

### 2.4. Custom guides manufacturing

**Figure 2:**
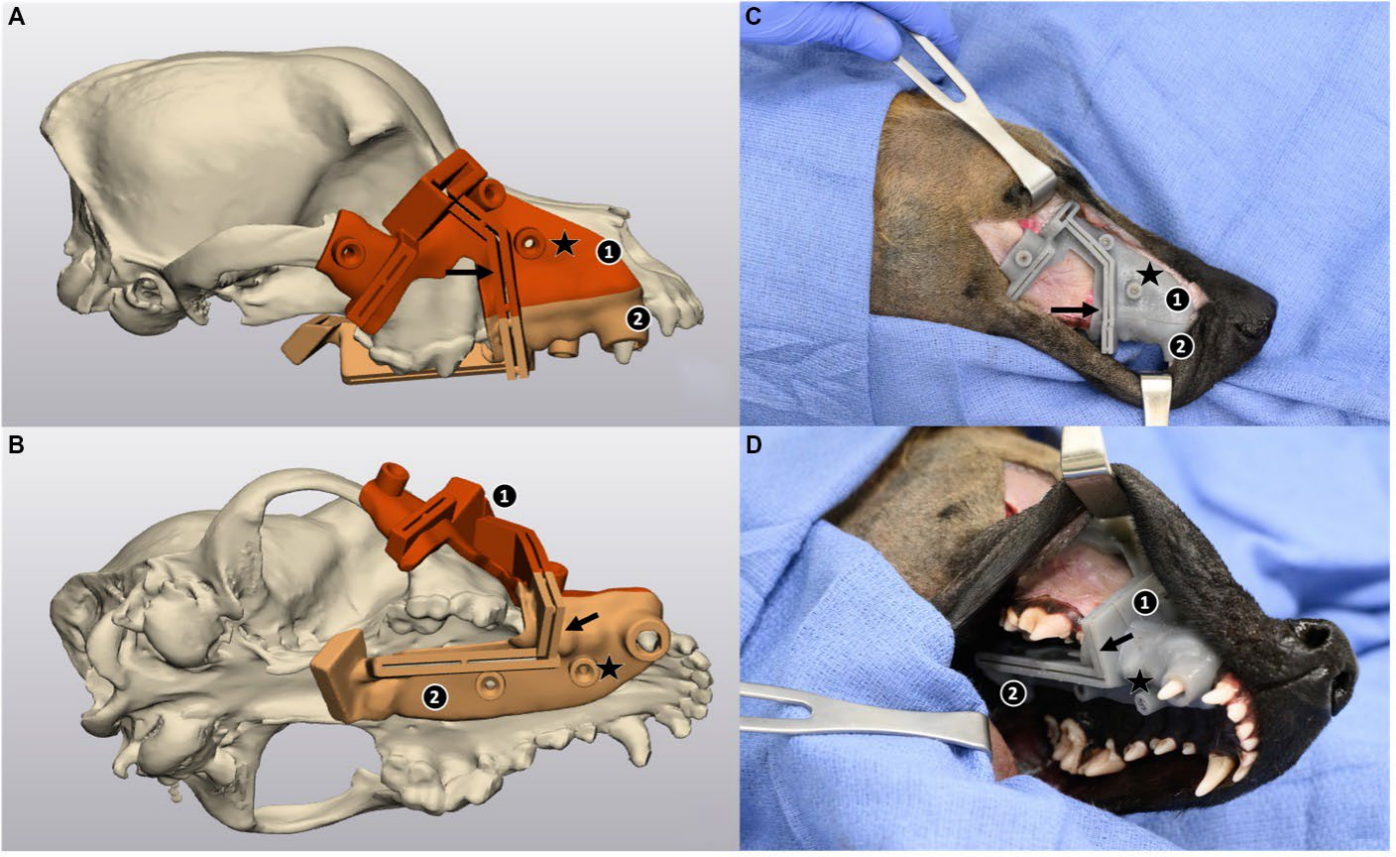
Three-dimensional model of a skull and computer-aided design (CAD) of a custom-made maxillectomy guide in lateral view **(A)** and ventrolateral view **(B)** in 3-Matic, and the corresponding views of the guide on a cadaver **(C,D)**. The guide is designed to recognize the contours of the skull, dental arch, gingiva, and palatine mucosa, and consists of 2 imbricating pieces (labeled 1 and 2), 5 drilling holes (star) to allow for the placement of cortical screws for stabilization, and 5 cutting grooves (arrow) guiding the 5 osteotomies. In **(C,D)** a combined dorsolateral and intraoral approach was performed to gain access to the maxilla. Screws have been placed to secure the guide in place.

Custom 3D-printed surgical guides were designed in 3-Matics software with the collaboration of an engineering undergraduate student (SN) at the Center for Additive Manufacturing and Logistics (CAMAL), for a total of 20 left and right sides of heads randomly allocated to treatment groups ESG and NSG. Each guide was made as two separate imbricating parts with a dorsal segment covering the orbit and dorsal maxilla and a ventral segment covering the caudal aspect of the dental quadrant and caudolateral palate as seen in Figure 2. Five drilling holes were added to secure the guide in place with 2.0 mm stainless steel screws (Arthrex Vet Systems, Naples, Florida, United States). The surgeon (MT) and surgery resident (AC) created the initial datum planes of the virtual cuts, and the cylinders at the location and orientation of the future screws as those require advanced knowledge of locoregional anatomy. The guide was then designed by the engineering student (SN) with strategical checkpoints along the design process at which times the CAD files were shared with the surgical team who reviewed the design before the engineering student could move on to the next step. The 3 checkpoints included a first review time before Boolean union of the parts, a second after trimming excess guide material, smoothening and planning the two-part sectioning of the guide, and a final checkpoint before printing. The cutting guides were printed in a stereolithography printer (Form 3; Formlabs, Sommerville, Massachusetts) with resin (Tough 1500; Formlabs, Sommerville, Massachusetts). Tough 1500 resin was chosen because it is considered biocompatible by the manufacturer as well as acceptable for steam autoclaving, gamma sterilization, and ethylene oxide sterilization.

### 2.5. Procedures

The head and procedure orders were randomized, and the experienced (MT) and novice (AC) surgeons performed the role of surgical assistant when not primarily involved in the procedure. Guidance for the guide placement or osteotomy was not provided to the novice from the experienced surgeon for the NSG treatment group, and *vice-versa*. All maxillectomy procedures were performed *via* a combined dorsolateral and intraoral approach as described by Lascelles et al. (19). This approach elevates the skin and nasolabialis muscle from the maxilla from the zygomatic arch to the rostrolateral aspect of the bone dorsal to the planned rostral margin; specificities of the guided procedure included exposure of the zygomatic arch and dorsolateral nose rostrally to the level of the canine, as well as the extension of the soft tissue dissection along the medial canthus of the eye to allow for guide placement. The guide was purposefully designed to allow placement without elevation of the gingiva or palatal tissue.

The freehand maxillectomy was performed as previously planned in the modeling software using the aforementioned anatomic landmarks (see Figure 1). The surgeon was allowed to visualize the model including the planned cuts in 3-Matics while drawing the planned resection on the cadaver head with a #15 scalpel blade to mark a thin cut line in the periosteum as one would visualize the CT images of the patients before marking the landmark of the cuts with an electrosurgical handpiece in a live procedure. For the guided osteotomy, the surgical guide was fitted to the head and secured in place with a total of five 2.0 mm cortical stainless-steel screws (Figure 2). The cuts were performed with a MicroAire oscillating saw (MicroAire, Charlottesville, Virginia) and a 0.6 mm Kerf blade for cuts 1 through 4; the orbital cut (5) was performed with a 15 mm × 2 mm osteotome and mallet. The guide was then removed after the screws had been retrieved to separate and extract the maxillectomy segment.

### 2.6. Quality assessment

For the ESG and NSG groups, the *ease of placement* for the guide was recorded as easy (able to fit the guide within 1 min without modification of the surgical approach), moderate (requiring modification of the surgical approach and/or able to fit the guide between 1 and 3 min), difficult (unable to fit or stabilize the guide without modifying the guide and/or able to fit the guide after 3 min).

Additionally, any instances of *guide failure*, including inability to fit the guide, cracking of the osteotome or saw groove, inability to secure the guide with screws, loose guide placement, or other failures were recorded.

For all three groups, the *quality of the cut* was recorded as high (smooth and straight cuts for all cuts and all cuts intersecting within 2 mm of their individual demarcation), moderate (one or two irregular cuts and/or cuts extending 2–5 mm beyond their intersection), or low (more than two irregular cuts and/or cuts extending >5 mm beyond their intersection).

### 2.7. Accuracy assessment

The linear deviation was calculated using the model-to-model distance extension in 3D Slicer from Kitware (Kitware Inc., Clifton Park, New York, United States). When the deviation was toward the excised segment, a positive value was assigned and when away from the excised segment, a negative value was assigned. Absolute values of these deviations were used to calculate the means.

**Figure 3:**
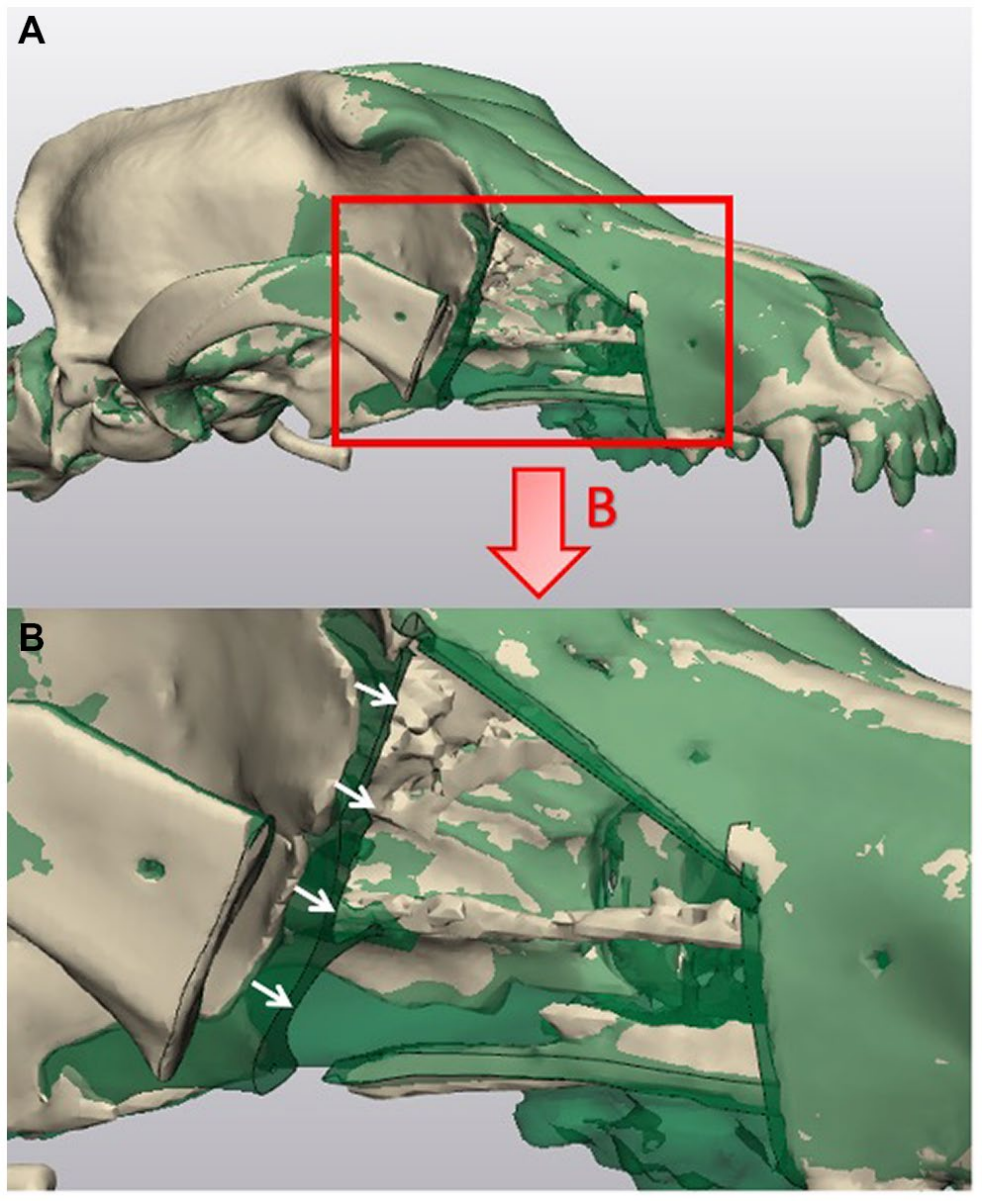
Representation of linear deviation evaluation between planned and performed maxillectomy. **(A)** Is a CAD superimposition of the post-maxillectomy skull with performed cuts (beige) and the virtual maxillectomy skull with planned cuts (green). **(B)** Is an inset of the zoomed in image of the superimposed skulls. The white arrows indicate an example of the distances that were measured using a cloud compare analysis to evaluate the mean linear deviation at the orbit cut.

Average linear deviation of the performed cuts from the planned cuts was used to measure accuracy between groups. The procedure used to obtain 3D models of the heads for planning the cuts was repeated with the skulls scanned post-maxillectomy (Figure 3). For guided subjects (ESG and NSG groups), an additional CT scan was performed with the guide secured in place prior to the osteotomy. This was included to investigate the origin of the deviation and establish if the variation observed between the planned and performed cuts was a result of an error at the time of guide placement (due to CAD/CAM and/or the individual placement considered *manufacturing/positioning error*) or a consequence of guide shifting during the osteotomy (considered *cutting error*).

To establish a comparison between *planned* (pre-op), *guided* (post-guide placement), and *performed* (post-maxillectomy) cuts in the ESG and NSG groups, the CT scans obtained at each step were overlaid in Geomagics to align them with each other. Local multipoint alignment was used to manually indicate prominent features on the skulls for the software to then align the models. In the ESF group, only planned and performed cuts were evaluated. Planes of the cuts were generated for each of the aligned skulls and guides in 3-Matic using the three-point method, where a skilled user picked the points based on cut alignment. In addition to this, the surfaces of each of the cuts were marked manually in 3-Matic and exported as STL files, along with the planes.

**Figure 4:**
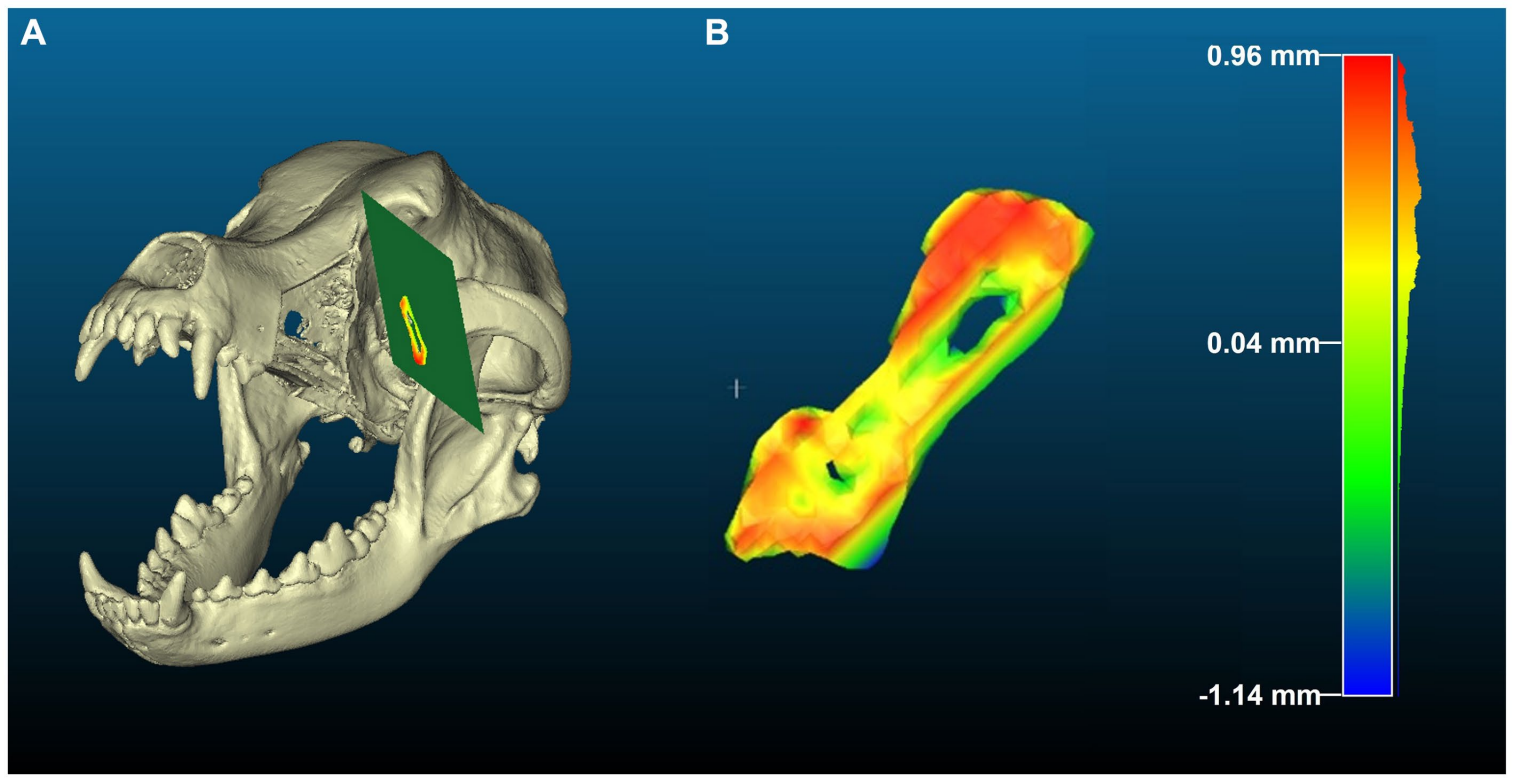
Heatmap generated using the “distance to primitive” function in cloud compare for cut 1 (orbit). **(A)** Demonstrates the planned cut plane in green and the performed cut in heatmap on the postoperative skull model. **(B)** Is a highlight of the cutting error observed along the orbit cut from planned to performed cut. In this case the cutting error was negative, or toward the defect, with the lowest to highest error pictured in warm (red) to cold (blue) colors, respectively. Scale bar indicates 15 mm. Color bar units are in millimeters.

Cloud Compare (open-source software), a 3D point cloud processing software, was used to calculate the deviation between the various generated planes and points on the cut surfaces obtained from CT scans superimposed (Figure 3), as reported in previous studies (20). For each cut, the surface STL was converted to a point cloud, and a primitive was generated by fitting a plane to the STL of the plane obtained from 3-Matic. The “distance to primitive” function was then used to obtain the distance of each of the points on the surface to the fit plane. Using this distribution, a histogram was generated and used for further analysis. Figure 4 shows an example of a heat map generated using the distances obtained for the zygomatic arch cut. The difference between the planned and performed cut were recorded as *total error* for each cut in all 3 treatment groups. The difference between the guided and performed cut was recorded for each cut only in the ESG and NSG groups, and considered *cutting error*. *The manufacturing*/*positioning errors* representing the difference between planned and guided cut was obtained by subtraction of the *cutting error* from the *total error* in the ESG and NSG groups.

### 2.8. Efficiency assessment

The portions of the maxillectomy procedures were divided in different time sequences to compare efficiency between ESG and ESF groups (reflecting on the use of the guide compared to freehand procedure) and between ESG and NSG groups (reflecting on the impact of surgical experience, while using the guide). The times required to model the corresponding head, and design and print each guide was also recorded as *CAD*/*CAM time*.

More specifically, for the ESF treatment group, the time to visually plan and mark the planned cuts on the cadaver head with a scalpel blade was recorded as *preparation time*. For the ESG and NSG groups, the *preparation time* was divided in *placement time* (time required to place the guide) and *securing time* (time required to secure the guide with screws), recorded separately.

The time to complete the osteotomy in the ESF treatment group was recorded as *freehand cut and removal time* and included the removal of the excised bone segment which clinically is performed concomitantly. In the ESG and NSG groups, the cutting and removal times were recorded separately as *guided cut* and *removal time* since the removal of the excised bone segment required prior removal of the guide.

The *total maxillectomy time* was recorded as the time from freehand planning or guide placement to osteotomy and removal of the excised bone segment. The surgical approach was not included in the time recorded. Accordingly, the *total maxillectomy time* for freehand subjects was an addition of *preparation* and *freehand cut and removal times.* The *total maxillectomy time* for guided subjects was the addition of the *placement, securing, guided cut, and guided removal times*.

### 2.9. Statistics

Based on a review of human literature regarding accuracy of 3D-printed custom-made surgical guides in maxillofacial, dental, and oncologic surgery, a mean linear deviation of 2 mm (+/−0.5) for both guided groups and 5 mm (+/−0.5) for ESF was expected (21–24). Using these expected values, a power analysis with an alpha of 0.05 and beta of 0.8 was performed and led to a minimum of 8 subjects per treatment group. Therefore, a total of 10 cadaver head sides was chosen as the number of subjects per group, which raised the total for 3 groups to 30 sides in 15 heads.

A Kruskal–Wallis test was performed to evaluate homogeneity across treatment groups in regards to head size.

The median and range was recorded for the total manufacturing time of the guides. The mean and standard deviations for times to plan freehand cuts, perform freehand cuts, place guide, secure guide, perform guided cuts, and remove the excised portions were recorded. The mean and standard deviations for linear deviation of each of the 5 cuts were calculated based on their absolute values, meaning that distances were examined without any consideration of which side of the designed cut they lay on. In cases where the performed cut intersected with the design cut, instead of the average then coming closer to 0 from having both positive and negative values, the side was ignored to estimate how much deviation from the design plane occurred.

Standard deviations were similarly calculated from the average absolute deviations and were compared to give insight into the consistency of various cuts. Comparisons were done *via t*-test or, where the normality assumption failed, Wilcoxon rank sum test on the square root of the average absolute deviation or on the untransformed standard deviation. The square root transformation was applied to improve the normality of the data.

For the accuracy statistical analyses, two sets of data were run: the primary set with all heads and cuts included, and an *exclusion set*. To maximize test subjects with the limited cadavers available, maxillectomies were performed on the left and right sides of the head, as described previously. For some cadaver heads, the anatomic structure of the remaining head was altered slightly once both left and right caudal maxilla were removed, creating some inexactitude in the measurement of the linear deviation. Those heads were excluded from analysis in the *exclusion set* of data.

## 3. Results

### 3.1. Specimens

The cadaver heads ranged in length of 17–32 cm from the maxillary canine to the caudal aspect of the occipital bone. Two heads were brachycephalic, two dolicephalic, and the remainder of the heads were mesocephalic. There was no significant difference in the head lengths between the groups (*p* = 0.5157 from a Kruskal–Wallis test). The cadaver head lengths are summarized in Table 1.

**Table 1:** Summary of the cadaver head lengths.

	Median (minimum-maximum) (cm)	Mean (standard deviation) (cm)	Head classification (brachycephalic/mesocephalic/dolicephalic)
ESF	23 (17–32)	23 (*±*4.5)	0/9/1
ESG	22 (17–32)	22.4 (*±*4.6)	1/8/1
NSG	21 (17–24)	20.8 (*±*2.4)	1/9/0

The length was recorded from the most ventral aspect of the maxillary canine to the caudal aspect of the occipital bone. There was no significant difference in the head lengths between the groups (*p* = 0.5157 from a Kruskal–Wallis test).

### 3.2. Quality assessment

#### 3.2.1. Ease of placement

Nine guides (9/10) were qualified as easy to place in the ESG group, and one guide (1/10) was graded as moderately easy. In the NSG group, 3/10 guides were considered easy to place, 6/10 moderately easy, and 1/10 difficult to place.

#### 3.2.2. Guide failure

Five instances of guide failure were observed, 4/5 in the ESG group and 1/5 in the NSG group; none of the failures yielded low quality cuts. Failures of the guide were observed as follows: 2/5 loosen securing screws, 1/5 osteotome slot crack, 1/5 saw slot crack, and 1/5 extension of the saw mark out of the cutting groove.

#### 3.2.3. Cut quality

Seven of thirty (23%) and 14/30 (47%) maxillectomy subjects had low or moderate quality cuts, respectively; distribution between groups is represented in Table 2. Of those with low or moderate quality cuts, 20/21 (95%) were related to the orbit cut. For the subject that had a moderate quality cut that did not occur with the osteotome, the jagged cut was located at the zygomatic arch. Nine subjects had incomplete cuts at the time of segment removal; 2 of those were observed in the ESF and ESG groups each, and 5 in the NSG group.

**Table 2:** Quality of the performed cuts.

Treatment group	Cut quality		
	Low	Moderate	High
ESF	2/10	4/10	4/10
ESG	1/10	5/10	4/10
NSG	4/10	5/10	1/10

Quality of the cut was recorded as high (smooth and straight cuts for all cuts and all cuts intersecting within 2 mm of their individual demarcation), moderate (one or two irregular cuts and/or cuts extending 2–5 mm beyond their intersection), or low (more than two irregular cuts and/or cuts extending >5 mm beyond their intersection).

### 3.3. Accuracy assessment

#### 3.3.1. Absolute linear deviation

**Figure 5:**
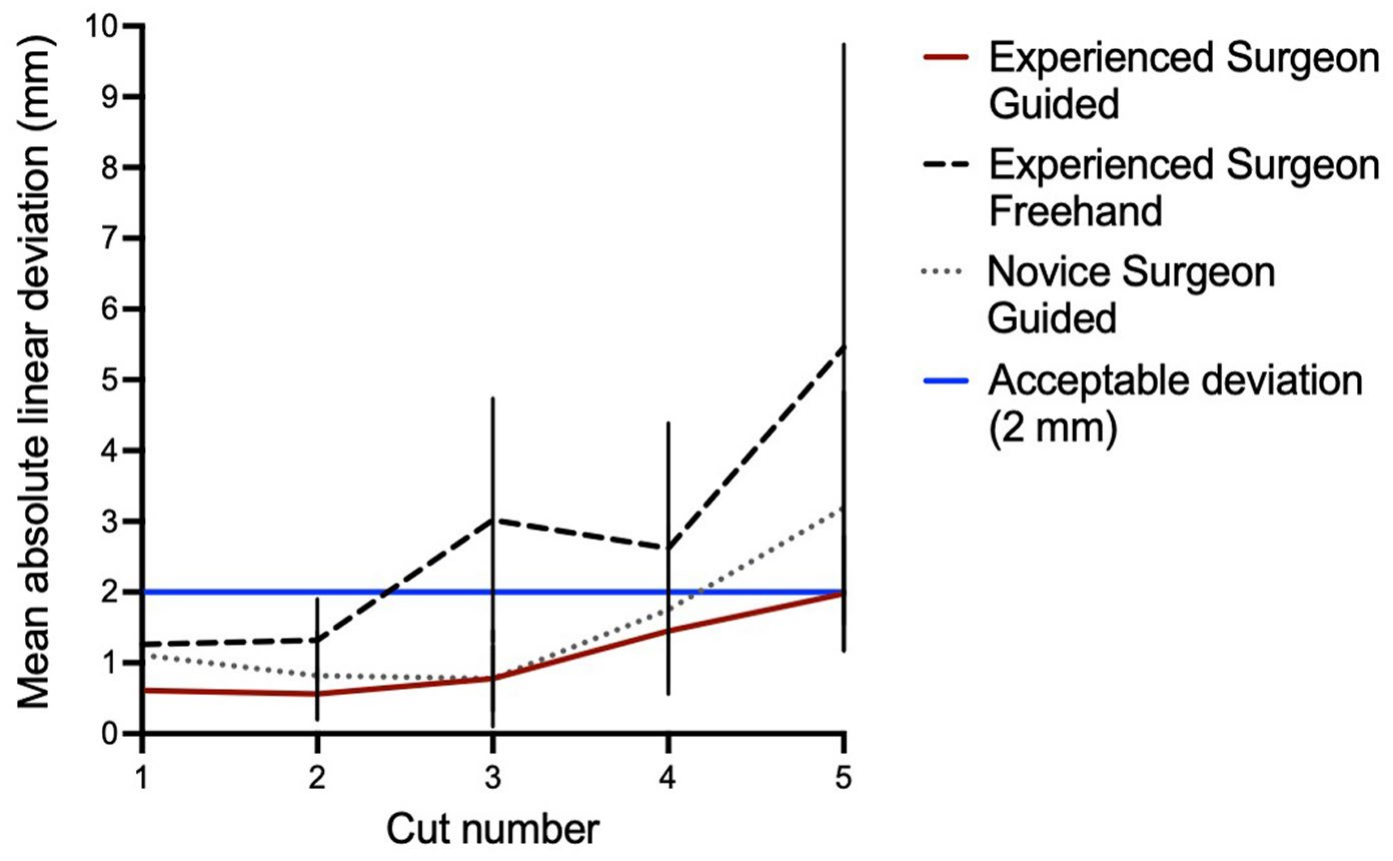
Linear deviation from planned to performed cut. All cuts for the experienced surgeon guided (ESG) fall within the acceptable deviation of 2 mm. Most of the novice surgeon guided (NSG) cuts fall within the acceptable deviation. Three of five cuts for the experienced surgeon freehand (ESF) are beyond the acceptable deviation (>2 mm).

The mean linear deviation from planned to performed cuts was overall lower in the ESG group compared to the ESF group, with a statistically significant difference for cuts 1 (*p =* 0.032), 2 (*p =* 0.003), 3 (*p <* 0.001), and 5 (*p =* 0.045) as shown in Table 3 and Figure 5. There was no significant difference in the mean linear deviation between ESG and NSG groups for any of the cuts. The greatest mean linear deviation from planned to performed cuts observed in the ESG, NSG, and ESF groups was 1.98 ± 0.81 mm, 3.19 ± 1.64 mm, and 5.46 ± 4.28 mm, respectively; all occurred at the orbit cut 5 (Table 3 and Figure 5).

**Table 3:** Summary values for average +/− SD absolute linear deviation from planned to performed cuts (mm).

Treatment group	Cut				
	1 (Zygomatic)	2 (Rostrolateral)	3 (Dorsolateral)	4 (Palatine)	5 (Orbit)
ESF	1.26 ± 0.76^A^	1.32 ± 0.58^A^	3.02 ± 1.72^AB^	2.62 ± 1.77	5.46 ± 4.28^A^
ESG	*0.61 (0.4, 0.73)^A^	0.56 ± 0.36^A^	0.78 ± 0.68^A^	1.45 ± 0.72	1.98 ± 0.81^A^
NSG	1.11 ± 0.7	0.82 ± 0.62	0.78 ± 0.46^B^	*1.75 (1.19, 2.35)	3.19 ± 1.64

Superscript (A, B) indicates significant differences between average absolute deviation for *p* < 0.05. Results are reported as median with quartiles for variables with significant deviation from normality and noted with an asterisk.

**Figure 6:**
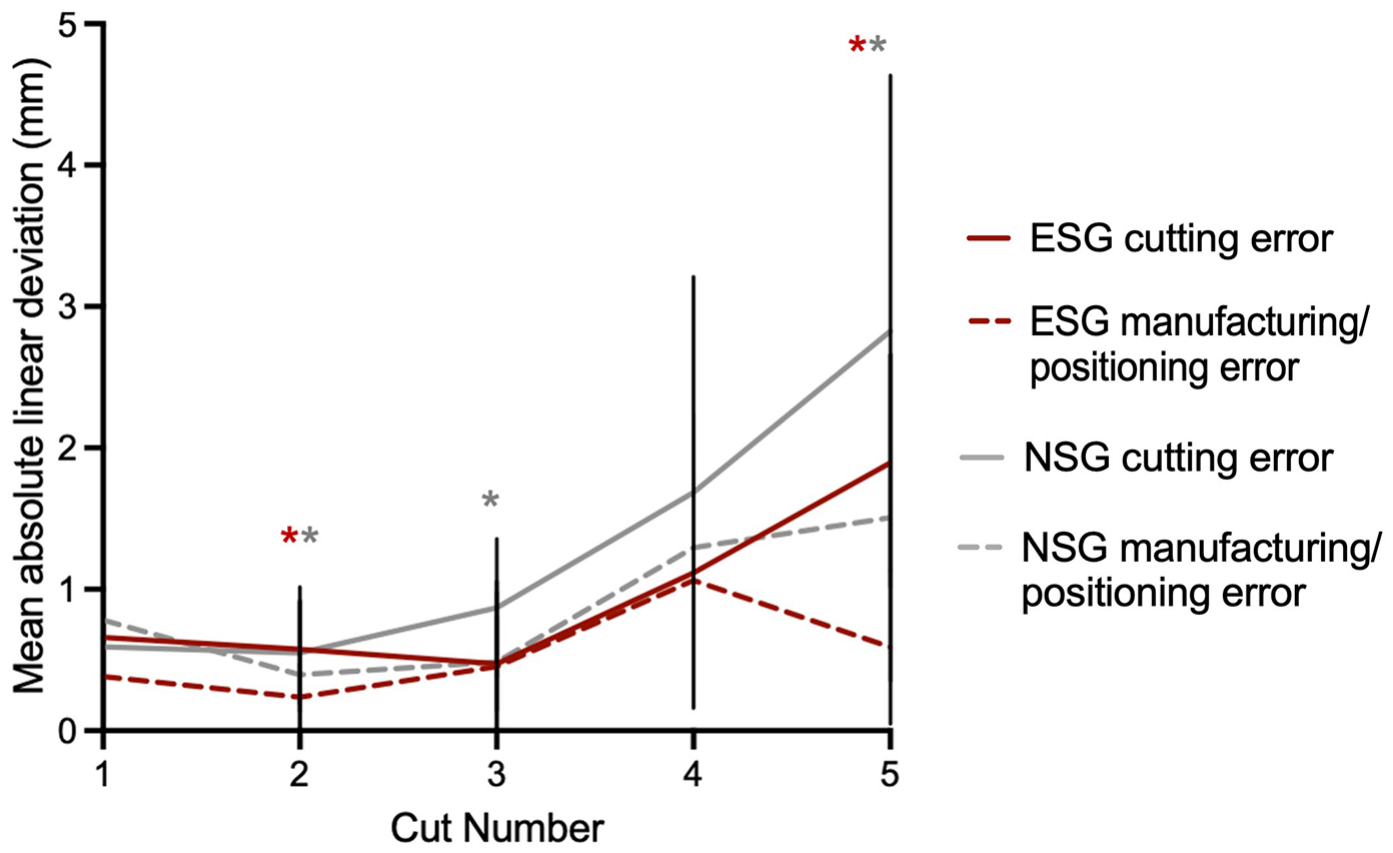
Origin of deviation observed with use of the guide. There is a trend toward the cutting error being more significant than the manufacturing/positioning error but that is not true for all cuts and only significant for the cuts with an asterisk. The asterisk indicates a significant difference between cutting error and manufacturing/positioning error with red for the experienced surgeon and gray for the novice. Although not statistically significant, there is a trend for the novice to introduce more cutting error than the experienced surgeon.

The stepwise breakdown of the mean linear deviation observed with the use of the guide is represented in Figure 6. For all cuts combined, there was a trend toward the cutting error being more significant than the manufacturing/positioning error, however this was statistically significant for ESG only for cuts 2 and 5 (*p* = 0.03 and *p* = 0.001, respectively), and for NSG for cuts 2, 3, and 5 (*p* = 0.021, *p* = 0.028, and *p* < 0.001, respectively). The total error was partially corrected from the positioning/manufacturing error to the cutting error due to difference in directions (positive vs. negative) in 43 and 48% of cuts for the NSG and ESG groups, respectively.

#### 3.3.2. Corrected values

Four heads were excluded in the *exclusion set* because of shifts in the skull anatomy post-maxillectomy suspected to be secondary to the removal of bilateral caudal maxilla. With those four heads excluded, there was no significant difference in mean linear deviation between the new values and the original data set.

#### 3.3.3. Consistency

The values for standard deviation between the planned and performed cuts are shown in Table 4. The standard deviation from planned to performed cut was significantly lower for the ESG group than the ESF group for cut 2 (*p* = 0.003) and cut 3 (*p* < 0.001). There was no significant difference in standard deviation from planned to performed cut and from guided to performed cut for the NSG group compared to the ESG group for any of the cuts. There was no difference in frequency of deviation in one direction versus the other for all cuts.

**Table 4:** Summary values for standard deviation from planned to performed cuts.

Treatment group	Cut				
	1	2	3	4	5
ESF	*0.12 (0.08, 0.16)	0.49 ± 0.47^AB^	1.25 ± 0.94^AB^	*0.15 (0.1, 0.41)^A^	3.28 ± 2.07
ESG	*0.08 (0.06, 0.14)	0.07 ± 0.05^B^	*0.04 (0.02, 0.09)^B^	*0.42 (0.19, 0.58)	*1.09 (0.41, 1.72)
NSG	0.1 ± 0.06	0.05 (0.05, 0.08)^A^	0.17 ± 0.15^A^	0.76 ± 0.58^A^	*0.85 (0.52, 2.57)

Superscript (A, B) indicates significant differences between average absolute deviation for *p* < 0.05. Results are reported as median with quartiles for variables. Significant deviation from normality is noted with an asterisk.

### 3.4. Efficiency assessment

#### 3.4.1. Manufacturing time

The median total *CAD*/*CAM time* for the ESG and NSG groups was 10.7 h, with a range of 9 to 16.6 h per guide including a median printing time of 4.75 h (range, 3.4 to 10.5 h).

#### 3.4.2. Preparation time

**Figure 7:**
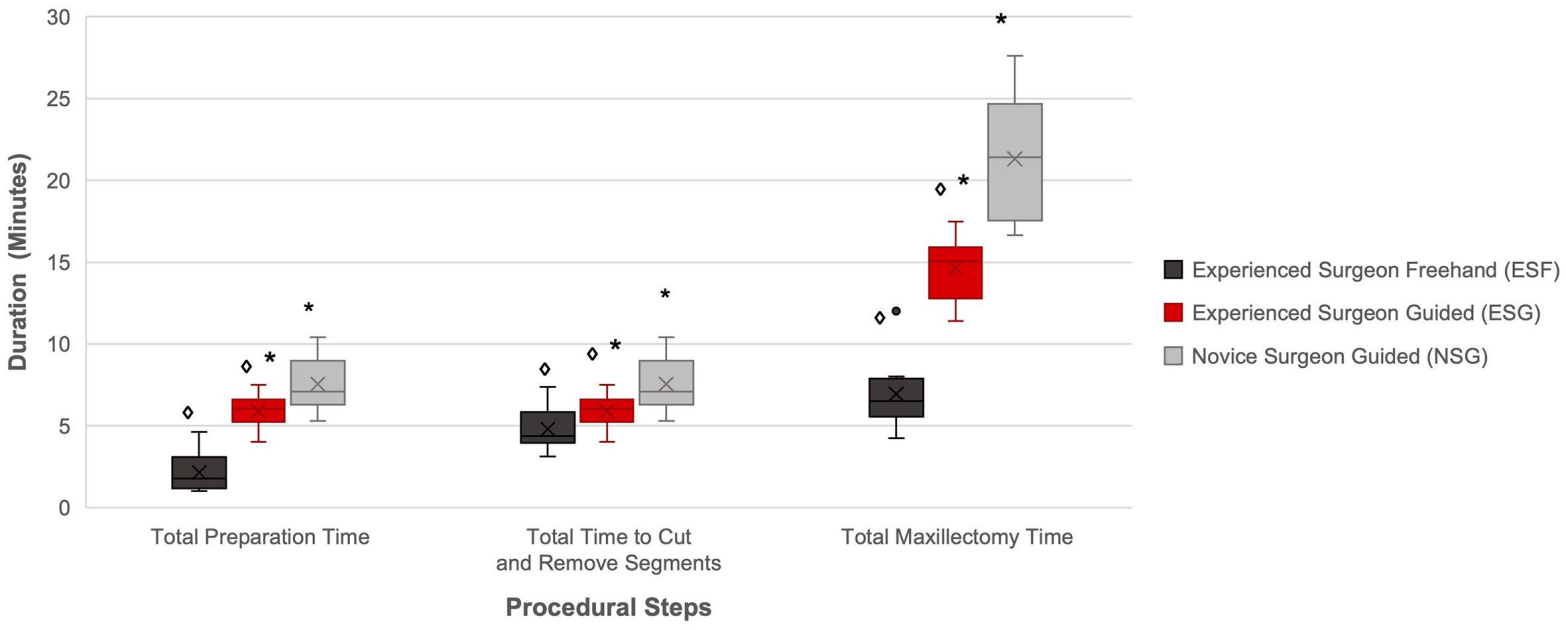
Comparison of total preparation time, cut and removal time, and total maxillectomy time per study group. The diamond and asterisks indicate significant differences (*p* < 0.05). There was a significant difference between ESF and ESG, and NSG and ESG for the total preparation time, total time to cut and remove the segment, and total maxillectomy time.

A significant difference was found in the *preparation time* between the ESG and ESF groups, with a median *preparation time* 3.38 time longer in the ESG group compared to the ESF group (*p <* 0.001) (Figure 7).

A significant difference was found in the *placement time* (*p =* 0.004) and the total *preparation time* (*p =* 0.023) between the ESG and NSG groups, with a median *placement time* and *preparation time* 2.3 and 1.2 time longer, respectively, for the NSG group compared to the ESG group (Figure 7). No significant difference was found in the median *securing time* between the ESG and NSG groups (*p =* 0.13).

#### 3.4.3. Cut and removal time

A significant difference was found in the cut and removal time between the ESG and ESF groups (*p <* 0.001), with a median *guided cut and removal time* 1.9 time longer than the *freehand cut and removal time* for the ESG group compared to the ESF group (Figure 7).

A significant difference was found in the *guided cut time* (*p <* 0.001), the *guided removal time* (*p =* 0.034), and in the total *guided cut and removal time* (*p <* 0.001) between the ESG and NSG groups, with the NSG group having a median *guided cut time, guided removal time, and guided cut and removal time* 1.5, 1.8, and 1.6 time longer, respectively, than the ESG group.

#### 3.4.4. Total maxillectomy time

A significant difference was found in the *total maxillectomy time* between the ESG and ESF groups (*p <* 0.001), with the ESG group having a median *total maxillectomy time* 2.3 time longer than the ESF group (Figure 7).

A significant difference was found in the *total maxillectomy time* between the ESG and NSG groups (*p <* 0.001), with the NSG group having a median *total maxillectomy time* 1.4 time longer than the ESG group (Figure 7).

## 4. Discussion

In this study, we successfully designed, manufactured and used 20 3D-printed custom-made caudal maxillectomy guides in canine cadavers. We found that the use of the guide did improve the overall accuracy of the procedure when evaluating the outcomes of the experienced surgeon compared to conventional freehand technique, and allowed the novice surgeon to reach similar accuracy. The use of the guide did, however, decrease the efficiency of the procedure compared to conventional freehand technique, and this was more pronounced with the novice surgeon compared to the experienced surgeon. Therefore, we completed our objectives and partially accepted our first and second hypotheses.

To the authors’ knowledge, this is the first veterinary study to specifically evaluate the accuracy of a custom-made 3D printed surgical guide for a caudal maxillectomy. A few veterinary studies have described the use of 3D printed cutting guides in combination with custom-made implants placement to reconstruct oncologic defects of the mandible, radius, tibia, and the skull (25–28). Improvements in accuracy have been reported with the use of surgical guides for pedicle screw placement in spinal surgery leading to a decreased risk of breaching the vertebral canal (29–31). In our study, an overall gain in accuracy was obtained with a global mean linear deviation improved from 2.74 mm to 1.08 mm with the use of the guide compared to the traditional freehand procedure for the experienced surgeon. This compares favorably with human literature reporting mean deviations of 1.17 mm and 2.49 mm from planned to achieved cuts with surgical guides, and ranges of 0.74 to 3.60 mm and 1.3 to 4.0 mm (11, 20, 23, 32). In comparison, the average deviation reported for freehand osteotomies when removing pelvic bone tumors in people is around 5 mm (13, 21). Most importantly, in our study, all cuts were within a mean linear deviation of 2 mm with the use of the guide while the most difficult cut along the orbit had a mean deviation superior to 5 mm without its use, similarly to previously reported freehand pelvic osteotomies (13, 21). This is particularly essential in an oncologic clinical scenario where the accuracy of oncologic margins could imply a difference between complete and incomplete tumor excision, leading to a higher risk of tumor recurrence and a potential need for adjuvant oncologic treatment. For most oral tumors, wide tumor excisions are planned with a margin of 1–2 cm of macroscopically normal tissue (33, 34). Based on those recommendations and previous papers evaluating accuracy of surgical guides, 2 mm was chosen as the threshold for a clinically acceptable surgical error (7, 20, 23, 32, 35, 36). Ultimately, achieving consistency in cutting accuracy is fundamental as one single largely deviated cut could be enough to lead to an incomplete excision. Our results show an improvement in the consistency for 2 out of 5 cuts with the use of the guide compared to the traditional freehand procedure. Overall, the use of the guide might help achieve more precise and more consistent osteotomy.

No significant difference in accuracy nor consistency was noted between the novice and the experienced surgeon suggesting that the 3D-printed custom-made surgical guide could provide assistance and reassurance to a less experienced surgeon performing a newly practiced procedure. Surgical approach and management of hemorrhage, however, are essential aspects of the procedure that are not exemplified by the guide. Few previous veterinary studies compared the accuracy obtained with the use of 3D-printed custom-made surgical guides from an experienced to a novice surgeon in corrective osteotomy and pedicle screw placement; no difference in accuracy was noted between the experienced and novice users in those scenarios (29, 37, 38). In the human literature, implant placement accuracy using 3D-printed custom-made surgical guides revealed no difference in accuracy between the experienced and novice surgeons in periodontal patients (39). This correlates to our findings and suggests that the guide represents a supportive surgical tool allowing a novice surgeon to perform a complex procedure without relying as intensely on traditional surgical mentorship. Additionally, the consistency of the procedure was similar between the novice and the experienced surgeon when using the cutting guide. This is in partial agreement with previous human studies as some report that custom guides allow novice surgeons to be as accurate as experienced surgeons and some suggests that experience still aids the user in obtaining higher accuracy even with the use of a guide (12, 39).

Part of our study design was also performed to measure the degree of error obtained at different steps of the guided procedure, as previous research has demonstrated that the total cumulative error is a sum of errors encountered during the CAD/CAM process, during the positioning of the implant, and during the actual cutting time (35, 40). The stepwise comparison between the planned, guided, and performed cuts revealed that the largest component of the total linear deviation originated from a cutting error, and suggests some existing micromotion after guide placement and during the performance of the osteotomy. The flexibility of the resin, the separation in 2 guide parts, the type of anatomic support (bone vs. mucosa vs. teeth), and the number of teeth included for support might all have played a role in this cumulative error (35, 40). Additionally, five of 20 guides showed some degree of failure (loosening of screws, osteotome or saw slot cracking, sawing outside of the groove) during use. Those failures may not represent relevant information to draw guidelines for guide use or manufacturing as the failure types reported were diverse and only one type of failure (screws loosening) occurred more than one time in this study. Overall, this is the first version of a caudal maxillectomy guide designed and tested in veterinary medicine; future prototyping might improve its outcome.

Finally, despite the noticeable improvement in accuracy reported in our study with use of the guide, the orbital cut carried the largest linear deviation in all groups and subjectively demonstrated a low to moderate cut quality in 20/30 (66%) of cases, all groups confounded. Overall, the cut quality along the orbit was higher for the experienced surgeon, with or without use of the guide, compared to the novice surgeon, which reflects on the difficulty of performing that part of the procedure. Its complexity is certainly related to the fact that the osteotomy is performed in a partially blind fashion, which is inherent to the procedure itself and the locoregional anatomy. One of the main purposes of our study was to facilitate that particular step by allowing the osteotome groove to guide the orbital cut, which was partially accomplished although our subjective impression remained mitigated. Improvement might come with some modifications in the guide design and use of different shapes and sizes of osteotomes.

Additionally, the overall efficiency of the procedure was lower compared to the traditional freehand technique. Therefore, our results compare unfavorably to human literature reviews of 3D-printed guides, which describe a decrease in intraoperative and operating room time in 80 and 46% of the cases, respectively (10, 41). Our results also compare unfavorably to a cadaveric veterinary study by Kim et al. evaluating the use of 3D-printed custom-made TPLO guides which reported a mean TPLO procedure time of 19 min with guide compared to 30 min without guide (42).

When breaking down the different procedural steps, the majority of the time difference observed for the experienced surgeon was found in the actual time required to perform the cut and remove the bone segment. This unfortunately also corresponds to the most critical step of the procedure where profuse hemorrhage can be encountered, thus the actual time of the procedure when an increase in efficiency would be beneficial. The choice made during our guide design to use screws instead of pins to improve guide stabilization might have been a trade off against efficiency, and replacing screws with pins could help placing and removing the guide faster. In addition, the cutting groove design did not allow to visualize the completion of the cuts at their intersections compared to the freehand procedure as the double cutting walls obscured the view; therefore, a larger number of incomplete cuts were noted in the guided groups, which was certainly responsible in part for the lack of efficiency. It is also possible that the efficiency demonstrated in the freehand group might have been overestimated by the use of cadavers. In live patients, surgeons generally experience profuse hemorrhage as the caudal maxillectomy is performed, unless the maxillary artery has already been ligated as previously described (43). Therefore, performing the maxillectomy and removing the segment in a live case scenario would likely take longer as blood obscures the field; the use of a guide could improve efficiency in that instance as the cuts could still be performed and finalized without absolute clear visualization.

Finally, the novice surgeon spent overall longer performing each step of the maxillectomy, and had a larger number of incomplete or subjectively low to moderate quality cuts. The increase in procedural time may have come from both the lack of familiarity with the procedure itself and with the use of a custom guide. Previous studies have demonstrated that experience with guides, even for the same individual, can improve the ability to position the guide (12). This was reflected as well in our efficiency and in our quality assessment as the placement of the guide was overall graded as more difficult for the novice group compared to the experienced surgeon. The cuts were also noted as incomplete in half of the cases for the novice group, which would have impacted the total time required to perform the cut and remove the maxillectomy segment. Therefore, although the use of a custom-made 3D printed guide might complement the surgical mentorship obtained, it certainly does not replace active supervision and might rather provide a comprehensive tool for initial practical exposure.

The main limitation of this study remains its cadaveric nature, and the fact that hemorrhage observed clinically may lower the efficiency and accuracy of the freehand procedure, therefore increasing the gap between the guided and traditional technique. The use of a guide may indeed aid in negating the effect of hemorrhage in a live patient because cuts could still be made even as blood obscures the field. Additionally, cadavers did not exhibit any pathology, and it is possible that the guide may not have the same fit with a large mass effect at the caudal maxilla. To accommodate for hypothetical large tumors, the guides were prophylactically designed with a large hollow center that would allow them to fit on the maxilla even in the instance where a tumor would surround the molars and/or last premolar teeth. Finally, the use and removal of left and right caudal maxilla to optimize cadaver usage and statistical power of our study design lead to four heads having a shift of the anatomy from an overall instability of the remaining skull. Even when excluding those four heads, however, the accuracy results were mostly identical and correlated with previous conclusions.

Another limitation of this study is the absence of comparison with a novice freehand group like previously reported by Bongers et al. (37) The goal of our study’s group design was to evaluate if a guide improved the accuracy and efficiency of a caudal maxillectomy for an experienced surgeon (comparison ESF to ESG groups) and to evaluate if the guide could allow a novice surgeon to perform the procedure with similar accuracy and efficiency of an experienced surgeon (NSG to ESG groups). Further work could be considered with a novice freehand group, however we decided against that evaluation considering that a caudal maxillectomy is a complicated procedure that would not be expected from a novice surgeon without completing a general surgical training program. A novice freehand group, however, could be considered to determine if the guide helps improve the accuracy and/or efficiency of the procedure for a novice surgeon, and if the improvement obtained with the guide is proportional to the difference provided to the experienced surgeon.

In addition to the guide efficiency in terms of use, the manufacturing time for the guides was lengthy. Some guides were printed as the sole objects on the printer plate and some were printed 2–3 guides to a plate. The number of objects on a printer plate greatly influences the duration of a print and therefore, the total manufacturing time that was reported in this study may not be realistic for the total manufacturing time required for a single surgical guide printed for a clinical case.

In conclusion, our novel 3D-printed custom-made cutting guide appears to improve the accuracy of the caudal maxillectomy for an experienced surgeon, and allows a novice surgeon to perform the procedure with similar accuracy. However, it is not evident that the efficiency of the procedure is improved with the guide. In freehand caudal maxillectomies, the orbit cut is frequently considered the most challenging and, for the guided procedures, continues to lead to the most inaccuracy. There is a trend toward greater accuracy with the guide for the orbit cut, however, and further improvements to a maxillectomy guide could facilitate a more accurate procedure.

## Data availability statement

The raw data supporting the conclusions of this article will be made available by the authors, without undue reservation.

## Ethics statement

Ethical review and approval was not required for the animal study because this study used cadavers from shelter animals euthanized for reasons unrelated to the study.

## Author contributions

AC, MT, SN, and OH contributed to conception and design of the study. AC, MT, SN, and SK designed the guides. AC and MT performed the cadaver work. SN and SK performed the analysis. AC and MT wrote the first draft of the manuscript. SK wrote sections of the manuscript. All authors contributed to the article and approved the submitted version.

## Funding

This work was funded by 2020 NCSU CVM Extramural Funding Incentive Grant ($10000).

## Conflict of interest

The authors declare that the research was conducted in the absence of any commercial or financial relationships that could be construed as a potential conflict of interest.

## Publisher’s note

All claims expressed in this article are solely those of the authors and do not necessarily represent those of their affiliated organizations, or those of the publisher, the editors and the reviewers. Any product that may be evaluated in this article, or claim that may be made by its manufacturer, is not guaranteed or endorsed by the publisher.
